# Phobia of the Supernatural: A Distinct but Poorly Recognized Specific Phobia With an Adverse Impact on Daily Living

**DOI:** 10.3389/fpsyt.2018.00590

**Published:** 2018-11-16

**Authors:** Ricardo de Oliveira-Souza

**Affiliations:** ^1^The D'Or Institute for Research and Education (IDOR), Rio de Janeiro, Brazil; ^2^Department of Neurology and Neuropsychiatry, The Federal University of the State of Rio de Janeiro, Rio de Janeiro, Brazil

**Keywords:** specific phobia, insomnia, fears, nocturnal anxiety, Anwesenheit, idea of presence

## Abstract

The psychological and psychiatric literature has seldom appreciated the clinical fact that fears of ghosts and kindred supernatural worries may be a cause of intense discomfort, poor sleep, and socio-occupational impairment. In the present article, this claim is illustrated by the clinical features of six patients who developed intense anxiety when they had to sleep alone at night. The fears were first noticed in childhood and persisted into adolescence and adulthood. At these times, they were overwhelmed by images of ghosts and haunted houses often experiencing a vivid impression that an immaterial being not perceivable by the ordinary senses was hovering around. Comorbidity with other phobias was the rule. Owing to shame and self-consciousness, the fears were seldom if ever discussed with healthcare professionals. The overall clinical and psychopathological picture was consistent with a diagnosis of a specific phobia. In a few cases, response to pharmacological treatment and cognitive-behavioral intervention has alleviated the symptoms. “Phobia of the supernatural” may be more common than usually thought. It must actively be sought for in patients complaining of poor sleep and daytime somnolence, and in patients with other types of phobia. The differential diagnosis of phobia of the supernatural includes nocturnal panic attacks, psychosis, other types of phobia that tend to occur during the night, dissociative states of sleep, dementia, and a few rare presentations of epilepsy. Systematic studies must be carried out to settle the neurobiological correlates of phobia of the supernatural as well as the possible benefit of different modalities of pharmacological and psychological treatment.

## Introduction

Few people would candidly admit fearing ghosts to the point of feeling uncomfortable if left home alone late at night. In fact, fears of the supernatural have only occasionally been a focus of attention in the clinical literature. In an early survey of fears in a large nonclinical sample, Hall allocated fears of the supernatural, such as of ghosts, spirits, and witches, to a specific category (1). He noted that the large majority of children probably pass at least a stage of fearing ghosts, but these fears tend to decline after adolescence.

Over the past 10 years, I have seen several patients whose lives were disturbed by their attitudes toward ghosts, spirits, and other supernatural “stuff.” Their stories revealed that they suffered from a type of phobia since childhood that eventually became disabling in critical sectors of their lives. The aim of the present report is to call attention to this possibly common and potentially treatable disorder, which is often concealed from clinical attention because of self-consciousness, shame, and misinformation. This is a strictly observational study, no patient having been subjected to any systematic research procedure or experimental intervention, therapeutic or otherwise. All patients consented in the publication of the present article through written informed consent. The D'Or Institute for Research & Education Scientific and Ethics Committee has approved this manuscript for publication in its present form.

## Report of cases

### Case 1

A 41-year-old physician was seen because of an episode of major depression from which he recovered in a few weeks with a daily dose of 150 mg of venlafaxine. When he returned, he casually asked whether the medication could have cured his fear of watching terror movies. He said that since he was a child he used to be “very impressed” by the supernatural. When he heard or read such tales, he could not sleep alone and went to his parents' bed. He felt ashamed and avoided being in touch with “such matters” over his entire life. After his first son was born, his wife would go to the baby's room to rock the infant to sleep. If she happened to fall asleep, he usually looked for an excuse to wake her up and bring her back to their room; otherwise, he would not fall asleep. His fears increased when he heard of supernatural themes. On these occasions, images related to the recent themes would pop up at night when he was alone; at those times, unexpected noises would bring to mind the recollection of a deceased relative or excerpts of popular terror movies or stories. When he was almost paralyzed by fear he felt as if there were someone nearby, yet he never hallucinated voices or visions. If, due to social circumstances, he could not avoid watching a horror movie at all, the most emotional scenes would later come to mind repeatedly, preventing him from sleeping. This effect persisted for a few nights and gradually returned to baseline levels. Thrillers or movies about catastrophes did not scare him the least. He only sensed that his fears could warrant professional attention after he became free of them. On questioning, we found that he also had a mild social phobia and that, since early childhood, his mother panicked whenever she saw a feathered bird.

### Case 2

A 46-year-old unmarried hotel attendant lost her father from chronic liver failure after a protracted illness. She was very upset because she would have to sell the apartment where she had lived with her parents since she was a teenager. The reason was that her mother had decided to move back to her hometown and she just could not stand the fear of living alone in their old apartment. She had painfully become aware of this a few days earlier, when her mother traveled for the weekend. She spent a whole night wandering around in the neighborhood and in a nightclub nearby because she felt scared at staying home alone. She had always been afraid of ghosts and supernatural themes, but she overcame her anxiety by asking her young nephews to sleep with her. More recently, she moved her bed to her parents' room “so that she might give her father a better assistance in case that he needed to get up in the middle of the night.” After he passed away, however, it became impossible for her to fall asleep, as vivid images of his funeral stubbornly came to her mind forcing her to turn on the lights and wake up her mother. She was treated with cognitive-behavioral therapy and sertraline plus alprazolam for several months. In 2 weeks, her sleep was continuous again, and she felt more confident to sleep in her room as long as her mother stayed home. After 3 months, she felt secure enough to live alone and to let her mother move away. She has now been treated for 3 years without a loss of therapeutic efficacy.

### Case 3

A 54-year-old lawyer intended to divorce his wife due to long-standing marital problems, but he wondered whether he would be bold enough to live alone. Since he was a teenager, he shared his room with an elder brother because he never managed to sleep alone. If left alone at night, he became fearful of ghosts and apparitions. He had a genuine interest in paranormal phenomena but could not read about them because it “sensitized” him and increased his fears at night. When his brother got married, he was desperate and got married less than a year later, after some embarrassing attempts to sleep with his parents. He had just graduated from college and admitted he never really loved his wife. He had a successful career but declined several opportunities to travel for work because he slept so badly in hotels that his performance was noticeably impaired. He could hardly bear staying alone in his office after hours, as he was increasingly disturbed by the feeling that someone was watching him over his back or just about to materialize before his eyes. He also complained of fear of closed places and of speaking in public. We did not succeed in controlling his symptoms due to gastrointestinal and cognitive adverse reactions to several drugs. He refused cognitive-behavioral therapy due to lack of time.

### Case 4

A 19-year-old college student was treated with fluoxetine for an episode of major depression of recent onset. Four months later, she reported that as her depressive symptoms subsided she also became free of fears that had tormented her since early childhood. Such fears were related to the feeling that spirits would break in her bedroom through the window. She never managed to sleep alone and shared a room with a younger sister. This seldom calmed her down, so she turned the lights on. This woke up her sister, who frequently became very upset. Eventually, her parents allowed her to sleep with them. If she were home alone in the evening, she turned all the lights on and never looked back because she was afraid that she might see ghosts right behind her. She avoided dark places and refused invitations to spend weekends with friends because she was afraid of being insomniac when away from home. She never watched terror movies. After 2 months on fluoxetine 40 mg/day, she gradually felt less fearful and became capable of sleeping alone in the dark, an improvement that has been sustained for 5 years.

### Case 5

A 63-year-old woman complained of “nightly attacks of fear”. Two years earlier, her husband passed away and she had to live alone for the first time in her life. Since that time, she needed increasing doses of benzodiazepines to calm her down and aid her falling asleep. In the evening, she felt that there was “someone in the living room.” This sensed presence was often her deceased husband but could be other entities unknown to her. These feelings were quite disturbing and embarrassing. On some occasions, she wet her bed in the middle of the night because she would not “dare” walk to the bathroom. She never woke up from sleep in panic, as typically happens in cases of nocturnal panic attacks. She denied being anxious or afraid of other specific situations. Her fears had worsened in the weeks before consultation due to the emergence of a major depressive episode. She felt increasingly hopeless and considered suicide. Before her husband's death, she described herself as an active and resourceful housewife, which was fully endorsed by her daughter. However, she was very shy, and always avoided speaking in public, for example, in church. She never hallucinated or developed delusions of any kind. She was a religious person and fully aware that her fears were unfounded. Since early childhood she would flee to her brother or sister's room in the night seeking relief from dreadful images of supernatural phenomena that came to her mind. She got married when she was 16 and lived a peaceful life with her husband. He worked as a truck driver and was often away from home several days at a time. On these occasions, she asked her neighbors to let one of their daughters to sleep with her. She was oriented to time and place and her general state of health was fair. She was treated with 1 mg of alprazolam and quickly improved. In 2 weeks, her phobic symptoms had dramatically diminished and she reassumed her previous level of autonomy. She has been doing well for the past 2 years as long as she complies with the alprazolam as originally prescribed.

### Case 6

The following account was written by a bright 11-year-old girl as a response to my request to describe her fears.

“*My fears are about supernatural stuff, even not believing in them. It's as if the fantasies of my mind make me imagine things that I've never really seen. Doors make me feel scared, regardless of whether they are shut or open, because I feel as if something is about to cross the doorway and chase me. When alone, I turn the lights on and look for a place from where I can see all the doors of my house. When I wake up in the middle of the night, which I do often, I immediately and involuntarily imagine dozens of horrible things, and usually end up thinking that something will snap in front of me out of the blue. I feel compelled to stay alert, so that I'm not caught by surprise if something does come by. I'm scared of darkness and never got used to it. One night it was raining, and my parents were out working. I panicked and called up my mother because I was overwhelmed by the feeling that a creepy being was just about to appear before my eyes and take hold of me. For the same reason, I don't sit in my bed with my feet hanging or placed on the floor, because I can't avoid expecting that hands from beneath my bed will grasp my heels and drag me down. These fears never come over if I'm not alone. Strangely, I seldom have nightmares*.”

This little girl is currently 29-years-old and about to finish a postgraduate course in History. She overcame her fears around puberty with no systematic treatment.

## Discussion

The cases herein presented are strikingly similar to those that were abridged by Hall at the end of the nineteenth century (1). One of his cases, a 23-year-old woman, “…was told [when she was 8] she might meet the spirit of her mother, who died when she was 2 days old; she longed to see her, but was so afraid that thereafter she would not look at her picture lest she should see her ghost, and everything about death and her mother became fearful (p. 230).”

Hall's and our cases meet current diagnostic criteria for a specific phobia., A specific phobia is currently defined as a persistent fear of clearly discernible, circumscribed objects or situations, exposure to which almost invariably provokes an immediate anxiety response (2). The phobic stimulus or situation is avoided or endured with dread. Moreover, the phobic anxiety interferes with the individual's life in significant ways. This “phobia of the supernatural” (PS), however, is not listed in most current inventories of phobic symptoms and is only obliquely mentioned in the literature. Consequently, its importance as a cause of social and occupational impairment has not duly been appreciated. For example, “superstitions such as fears of houses said to be haunted by ghosts” were given as an example of normal fears in children (3). The few studies that included fears of ghosts and related spooky entities in their inventories concur that they tend to decline during adolescence or soon thereafter (4, 5). Although these authors also note that some fears may persist into adulthood, the implications of fearing ghosts in everyday life have not so far been examined.

## The syndrome of phobia of the supernatural

Our cases demonstrate that the persistence of fears of ghosts may lead to social and familial impairments as well as to financial losses. In agreement with the aforementioned authors, PS had an early onset in our cases. Most of them got relief from their fears in their siblings' or parents' room when they were young. As is usually the case in patients with phobic symptoms, other types of phobia were commonly associated with PS. PS is typically elicited by staying alone indoors at night. In these situations, anxiety is associated with “Anwesenheit” (6), or “the idea of a presence” (7), that is, the vivid impression that some immaterial being who cannot be apprehended by the ordinary senses is hovering around. Anwesenheit has been described in normal people (8), but it is a constant experience in our cases. When they had to sleep alone for whatever reason, the simple mention of words related to death or the afterlife, such as “graveyard,” “burial,” “satanic cult,” evoked concerns about anxiety and bad sleep in the ensuing nights. The silence of the wee hours added to the dread, every ambient noise increasing the fear. PS abates almost instantly if they have someone at their side. In some cases, a pet may have a soothing effect as well; turning the lights on, watching television, or going out may likewise attenuate the symptoms. That PS is specific to supernatural themes is shown by the absence of anxiety involving darkness, tragic scenes, natural disasters or bloody scenes, as long as they do not evolve into “beyond the grave” topics.

Sleep impairment with subsequent daytime somnolence and decrease in productivity are major symptoms of PS. Sleep onset is delayed and when anxiety is eventually overcome by tiredness, sleep is fragmentary and haunted by dreadful images and thoughts. Nightmares of witchcraft and allied supernatural themes are common. Therefore, specific questions addressing PS should be included in inventories for the assessment of poor sleep and insomnia. The differential diagnosis of phobia of the supernatural includes sleep terror, other phobias (e.g., phobia of darkness unrelated to supernatural ideation), epilepsy (9), dissociative states of sleep (10), and dementia (11). Nocturnal panic attacks should thoroughly be differentiated from PS (12). Such attacks usually awaken the patient from NREM sleep between 01:30 and 03:30 a.m. and may be the primary manifestation in a subset of patients with a diagnosis of anxiety disorder (13). These diagnoses are easily ruled out by careful history taking and symptom characterization in each case.

Patients with PS are usually reluctant to acknowledge the real motive beneath their nocturnal anxiety because they tacitly assume that their fears are part of their psychological makeup or too weird to be mentioned. Therefore, PS will be overlooked if not specifically sought for. A couple of questions such as “are you afraid of ghosts?” or “are you afraid of staying home alone at night? [If so] Why?” will expose the diagnosis in virtually all cases. Until systematic studies on the response of PS to specific therapies are conducted, a trial of antidepressants or benzodiazepines and cognitive-behavioral therapy may pay off. Letting them know that fear of ghosts is a symptom of a discrete neuropsychiatric disorder that may benefit from treatment is reassuring and often a first step on the way to rational management.

Future research is needed to elucidate the neural correlates of PS. There is good evidence that the amygdala and its connections with other structures of the basal forebrain and the cerebral cortex are critical for the experience of fear, suggesting that the amygdala is an essential pathophysiological node underlying at least a few types of phobia. Both functional neuroimaging (14) and lesion (15, 16) studies may provide relevant information on the neural concomitants of the experience of dread, and, more to the point, on the haunting experience of sensing the presence of ghosts and other immaterial entities (17).

## Author contributions

The author confirms being the sole contributor of this work and has approved it for publication.

### Conflict of interest statement

The author declares that the research was conducted in the absence of any commercial or financial relationships that could be construed as a potential conflict of interest.
